# The Pace of Cognitive Aging in Older Adults Without a Neurocognitive Disorder

**DOI:** 10.1093/geroni/igaf122.537

**Published:** 2025-12-31

**Authors:** Zachary Kunicki, Emma Nichols, Alyssa De Vito, Cyrus Kosar, Adea Rich, Emily Briceño, Alden Gross, Richard Jones

**Affiliations:** Warren Alpert Medical of Brown University, Providence, Rhode Island, United States; University of Southern California, Los Angeles, California, United States; Brown University, Providence, Rhode Island, United States; Brown University, Providence, Rhode Island, United States; Columbia University, New York, New York, United States; University of Michigan Medical School, Ann Arbor, Michigan, United States; Johns Hopkins Bloomberg School of Public Health, Baltimore, Maryland, United States; Brown University, Providence, Rhode Island, United States

## Abstract

The pace of cognitive change without a neurocognitive disorder is one of the major questions in cognitive aging. The Children of the Depression Age (CODA) cohort of the Health and Retirement Study (HRS) is ideal for studying cognitive aging because it has a long follow-up (22 years) and a narrow age range at baseline (67-74 years). We analyzed data on delayed word recall performance in HRS-CODA (N = 2,295), gathered every other year during the follow-up period. Using latent growth curve modeling and treating delayed recall as a categorical outcome, we found a quadratic model showed better fit to the data than a linear model, with no strong evidence for a practice and retest effect. When calculating the pace of normative aging, our results suggested normative (defined as the absence of a dementia diagnosis over the follow-up period) memory decline is about -0.05 standard deviations per year (SD/y), but is better characterized by age specific estimates of -0.04 SD/y, -0.10 SD/y, and -0.15 SD/y for individuals in their 70s, 80s, and 90s, respectively. These results would suggest that memory decline, in the absence of a recognized dementia and without a confounding of baseline age differences and longitudinal age changes, would be present but almost imperceptible to an individual in their eighth decade, but noticeable in their ninth and quite impairing in their tenth decade.

